# Widespread activation of immunity and pro‐inflammatory programs in peripheral blood leukocytes of HIV‐infected patients with impaired lung gas exchange

**DOI:** 10.14814/phy2.12756

**Published:** 2016-04-25

**Authors:** Kristina Crothers, Irina Petrache, Cherry Wongtrakool, Patty J. Lee, Lynn M. Schnapp, Sina A. Gharib

**Affiliations:** ^1^ Division of Pulmonary & Critical Care MedicineHarborview Medical CenterUniversity of WashingtonSeattleWashington; ^2^ Division of Pulmonary & Critical Care MedicineNational Jewish HealthDenverColorado; ^3^ Pulmonary SectionDepartment of Veterans Affairs Medical CenterDecaturGeorgia; ^4^ Division of PulmonaryAllergy, Critical Care, & Sleep MedicineEmory UniversityAtlantaGeorgia; ^5^ Division of Pulmonary & Critical Care MedicineYale UniversityNew HavenConnecticut; ^6^ Division of Pulmonary & Critical Care MedicineMedical University of South CarolinaCharlestonSouth Carolina

**Keywords:** Human immunodeficiency virus, lung diffusing capacity, microarray, network

## Abstract

HIV infection is associated with impaired lung gas transfer as indicated by a low diffusing capacity (DLCO), but the mechanisms are not well understood. We hypothesized that HIV‐associated gas exchange impairment is indicative of system‐wide perturbations that could be reflected by alterations in peripheral blood leukocyte (PBL) gene expression. Forty HIV‐infected (HIV ^+^) and uninfected (HIV ^–^) men with preserved versus low DLCO were enrolled. All subjects were current smokers and those with acute illness, lung diseases other than COPD or asthma were excluded. Total RNA was extracted from PBLs and hybridized to whole‐genome microarrays. Gene set enrichment analysis (GSEA) was performed between HIV ^+^ versus HIV ^–^ subjects with preserved DLCO and those with low DLCO to identify differentially activated pathways. Using pathway‐based analyses, we found that in subjects with preserved DLCO, HIV infection is associated with activation of processes involved in immunity, cell cycle, and apoptosis. Applying a similar analysis to subjects with low DLCO, we identified a much broader repertoire of pro‐inflammatory and immune‐related pathways in HIV ^+^ patients relative to HIV ^–^ subjects, with up‐regulation of multiple interleukin pathways, interferon signaling, and toll‐like receptor signaling. We confirmed elevated circulating levels of IL‐6 in HIV ^+^ patients with low DLCO relative to the other groups. Our findings reveal that PBLs of subjects with HIV infection and low DLCO are distinguished by widespread enrichment of immuno‐inflammatory programs. Activation of these pathways may alter the biology of circulating leukocytes and play a role in the pathogenesis of HIV‐associated gas exchange impairment.

## Introduction

Pulmonary complications remain a significant cause of morbidity and mortality among HIV‐infected (HIV^+^) individuals despite the advent of combination antiretroviral therapy (ART). While opportunistic pulmonary infections have declined, the incidence of noninfectious, chronic lung diseases has increased and is expected to grow with the increasing lifespan of patients. One of the most common pulmonary manifestations of HIV infection is impaired gas exchange in the lung as reflected by a low diffusing capacity of carbon monoxide (DLCO) (Crothers et al. 2013; Fitzpatrick et al. 2013). Although low DLCO was first noted in HIV^+^ individuals prior to the era of combination ART (Mitchell et al. 1992; Rosen et al. 1995), HIV^+^ patients remain significantly more likely to have moderately to severely reduced DLCO despite therapy when compared to HIV^–^ subjects, even after adjustments for smoking and other risk factors (Crothers et al. 2013). Impaired DLCO can reflect lung pathology such as the presence of emphysema, fibrosis, or pulmonary hypertension, or can occur as a consequence of other conditions associated with HIV, including *Pneumocystis* pneumonia or injection drug use. In epidemiologic studies, emphysema appears to be the most common lung disease associated with low DLCO in HIV^+^ patients (Diaz et al. 1992, 2000).

The mechanisms leading to impaired diffusing capacity in HIV infection are poorly understood. HIV infection is associated with systemic chronic inflammation, endothelial dysfunction, altered coagulation, and immune activation – processes that are tightly linked to increased morbidity and early mortality in HIV^+^ patients, even among those on effective ART (Kuller et al. 2008; Baker et al. 2010, 2011; Dubé and Sattler 2010; Neuhaus et al. 2010; Sandler et al. 2011). Dysregulation of these pathways may injure the lung, causing abnormal gas exchange. Indeed, we have demonstrated that chronic immune activation, as reflected by elevated levels of circulating soluble CD14 (sCD14), a component of the innate immune system, is associated with emphysema in HIV^+^ individuals (Attia et al. 2014).

To gain a better understanding of putative processes involved in impaired gas exchange during HIV infection, we surveyed the transcriptional landscape of circulating peripheral blood leukocytes (PBLs) in HIV^+^ and HIV^–^ subjects with preserved or reduced DLCO. Since infection with HIV by itself can cause widespread alterations in leukocyte gene expression, we compared relative enrichment of pathways between HIV^+^ versus HIV^–^ individuals with preserved DLCO against processes enriched in HIV^+^ versus HIV^–^ subjects with low DLCO. We hypothesized that although HIV infection can activate a common core of transcriptional programs in PBLs, some processes may be distinct between subjects with preserved lung diffusing capacity versus those with low DLCO. Identifying these pathways can provide novel insights into the pathogenesis of HIV‐associated impairment in pulmonary gas exchange.

## Materials and Methods

### Study sample

We studied a total of 40 HIV^+^ and HIV^−^ men with preserved versus low DLCO, who were enrolled in the Examinations of HIV Associated Lung Emphysema (EXHALE) study, a pulmonary‐focused component of the Veterans Aging Cohort Study (VACS) (Justice et al. 2006a). EXHALE was an observational, longitudinal multicenter study conducted at four of the eight Veterans Affairs (VA) Medical Centers (VAMC) participating in VACS, and has been described previously (Attia et al. 2014; Campo et al. 2014). Individuals with a history of lung diseases other than COPD or asthma were excluded, as were patients with acute respiratory infections or illness in the 4 weeks prior to the baseline measurements. Participants were enrolled between 2009 and 2012. All subjects included in this analysis were current smokers. Institutional Review Boards at all locations approved this study, and participants provided written informed consent.

### Clinical data collection

Baseline study procedures for EXHALE that were included in these analyses consisted of a questionnaire, pulmonary function testing (PFT), and chest computed tomography (CT) scan. At study entry, all participants self‐completed a questionnaire that consisted of a standardized assessment of smoking and drug use (Comstock et al. 1979). Demographic and pharmacy data, laboratory values, and diagnostic codes (ICD‐9) for existent medical conditions were obtained via the VA national electronic medical records. Variables included age, gender, race, and ART. Clinical laboratories including hemoglobin, CD4 cell count, and plasma HIV RNA level were obtained within 6 months of enrollment. A peripheral white blood cell count (WBC) and differential was also obtained from clinical records; median time between WBC count and the research blood draw date was 0 days (interquartile range, −27 to 30 days).

Pulmonary function tests were performed according to American Thoracic Society criteria (Standardization of Spirometry, 1995; Miller et al. 2005). Diffusing capacity was measured by transfer of carbon monoxide (DLCO) by single breath method (Macintyre et al. 2005). PFTs were obtained by certified, trained respiratory technicians in the clinical pulmonary function laboratories at the associated medical center. Fixed airflow obstruction consistent with COPD was defined primarily as a ratio of the post‐bronchodilator forced expiratory volume in one‐second (FEV_1_) to forced vital capacity (FVC) below 70%. Predicted normal values for spirometry were determined using Hankinson formulas, and for DLCO using Neas formulas (Neas and Schwartz 1996; Hankinson et al. 1999). Both these formulas include adjustments for age, gender, race/ethnicity, and height; percent predicted DLCO was also corrected for hemoglobin concentration. Preserved DLCO was defined as greater than 60% of predicted value and low DLCO was defined as ≤60% predicted value. We chose a cutoff of ≤60% of predicted normal for a low DLCO in order to reflect a more severe phenotype of disease, reflective of moderate to severe impairment. In addition, a DLCO ≤60% avoids misclassification from a mildly reduced DLCO that could be due to active smoking. Because we required that all subjects in this analysis were current smokers, insufficient numbers of current smokers had a DLCO that was >80% of predicted; while we identified as many with a normal DLCO as possible, we also included several subjects in the preserved DLCO group with a DLCO between 60% and 80% of predicted. PFT results were interpreted blinded to HIV status, and severity of impairment graded according to the ERS/ATS 2005 recommendations (Pellegrino et al. 2005).

Noncontrast chest CT scans were obtained in subjects at baseline. A standardized interpretation was performed by a radiologist blinded to the HIV status and other clinical characteristics of the subjects. Findings were classified as per Fleischner Society definitions (Hansell et al. 2008). Severity of emphysema was scored using a semiquantitative scale (Wilson et al. 2008; Attia et al. 2014), and pulmonary artery enlargement, measured at the level of bifurcation, was defined based on a main pulmonary artery to aorta diameter of greater than one.

### Microarray data analysis

Peripheral blood was collected from each EXHALE participant at study enrollment. In the 40 subjects identified for these analyses, total RNA from each sample was isolated using Paxgene Blood RNA kits (Qiagen, Valencia, CA), labeled, and hybridized to a PrimeView Human Gene Expression microarray (Affymetrix, Santa Clara, CA) according the manufacturer's protocol. The PrimeView microarray is comprised of more than 36,000 transcripts mapping to over 20,000 unique genes. After image processing, microarrays were background adjusted and quantile normalized across all subjects using robust multi‐array averaging (RMA) (Bolstad et al. 2003). One sample did not meet strict microarray hybridization quality control thresholds and was excluded from further analysis. The remaining 39 samples were comprised of four subject groups: 1. HIV^–^, preserved DLCO (*N* = 9); 2. HIV^+^, preserved DLCO (*N* = 9); 3. HIV^–^, low DLCO (*N* = 11); 4. HIV^+^, low DLCO (*N* = 10). Detailed microarray experiment description meeting Minimum Information About a Microarray Experiment (MIAME) requirements has been deposited at Gene Expression Omnibus (http://www.ncbi.nlm.nih.gov/geo, GSE76403).

We applied Gene Set Enrichment Analysis (GSEA) to identify and compare pathways differentially enriched between HIV^+^ and HIV^–^ subjects with preserved DLCO versus those with low DLCO (Subramanian et al. 2005). Rather than focusing on differential changes in a single gene, GSEA statistically assesses whether a large subset of genes mapping to a given pathway (known as “leading edge”) are up‐regulated in the phenotype of interest. GSEA calculates an enrichment score (ES) for overrepresentation of up‐regulated genes for a given gene set and then applies a random permutation analysis to estimate significance level of ES and adjust for multiple hypothesis testing. We performed 1000 permutations and selected a highly stringent false discovery rate (FDR) threshold <0.01 to designate statistically significant enrichment. To maximize biological relevance, we focused on gene sets derived from 1315 well‐curated canonical pathways compiled from multiple resources including Kyoto Encyclopedia of Genes and Genomes (KEGG), Biocarta, and Reactome among others.

We applied GSEA to HIV^+^ versus HIV^–^ subjects with preserved DLCO (group 1 vs. group 2) to assess the role of HIV infection on activation of PBL transcriptional programs. Next, we performed GSEA on HIV^+^ versus HIV^–^ subjects with low DLCO (group 3 vs. group 4) to identify pathways differentially enriched as a consequence of HIV infection in patients with impaired lung gas exchange. Relationships among enriched gene sets were charted based on degree of shared genes using a network‐based visualization method known as Enrichment Map (www.baderlab.org/Software/EnrichmentMap/) implemented within the Cytoscape environment (Cline et al. 2007; Merico et al. 2010). To further assess the influence of HIV infection on altering pathway enrichment in circulating leukocytes, we performed GSEA in HIV^–^ subjects with preserved versus low DLCO (group 1 vs. group 3), and in HIV^+^ patients with preserved versus low DLCO (group 2 vs. group 4).

### Circulating IL‐6 and sCD14 measurements

Serum IL‐6 levels were measured in all subjects using a chemiluminescent immunoassay (QuantiGlo IL‐6 immunoassay, R&D Systems, Minneapolis, MN). Calibration was performed by the manufacturer and is traceable to National Institute for Biological Standards and Control 89/548 (IU/mL). Soluble CD14 (sCD14) was measured with an enzyme‐linked immunosorbent assay (Quantikine sCD14 Immunoassay, R&D Systems) with a detectable range of 40–3200 ng/mL, using a standard 200‐fold sample dilution. Statistical differences in cytokine levels between groups were assessed using two‐tailed *t*‐test, and across all four groups using one‐way analyses of variance (ANOVA) (GraphPad Software, La Jolla, CA).

## Results

### Subject characteristics

Table 1 summarizes the cohort characteristics. Subjects with low DLCO were slightly older and more likely to be African‐American. Although all subjects were current smokers, the HIV^+^ patients tended to have a heavier smoking history. Most HIV^+^ subjects were on ART and had suppressed HIV viral loads (<50 copies/ml). CD4 counts were significantly lower in HIV^+^ patients with reduced DLCO compared to those with preserved DLCO. Pulmonary function tests demonstrated a significantly lower percent predicted FEV_1_ and FVC in subjects with low DLCO, although values were within the normal range. HIV^+^ patients were more likely to have evidence of pulmonary artery enlargement on chest CT scan, regardless of DLCO. A greater proportion of HIV^+^ subjects had radiographic evidence for emphysema, particularly those with low DLCO. The peripheral WBC count was significantly different across groups, with lowest values in patients with HIV infection and a low DLCO.

**Table 1:** Patient characteristics, lung function measures, and peripheral blood counts

Characteristic	HIV^+^ low DLCO (*n* = 10)	HIV^+^ preserved DLCO (*n* = 9)	HIV^−^ low DLCO (*n* = 11)	HIV^−^ preserved DLCO (*n* = 9)	*P*‐value^a^
Age, years	52 (51–57)	49 (40–53)	50 (48–57)	48 (46–50)	0.03
Black race	90%	67%	91%	56%	0.3
Smoking pack‐years	35 (21–40)	28 (10–34)	20 (16–26)	12 (8–31)	0.2
CD4 cell count, cells/mm^3^	310 (241–323)	507 (441–707)	–	–	0.004
HIV RNA copies/mL^b^	<50	<50	–	–	0.5
On ART	70%	100%	–	–	0.2
Pulmonary function tests					
FEV_1_, % predicted	79 (72–88)	109 (105–110)	79 (70–92)	96 (89–103)	0.002
FVC, % predicted	99 (95–103)	85 (77–95)	105 (102–115)	81 (74–87)	0.0008
FEV_1_/FVC, %	75 (70–81)	79 (75–82)	80 (76–82)	75 (71–80)	0.3
DLCO, % predicted	39 (37–40)	83 (71–84)	40 (35–43)	74 (67–89)	0.0001
Chest CT scan data					
Any radiographic emphysema	89%	56%	70%	33%	0.09
Pulmonary artery enlargement	22%	56%	10%	11%	0.1
WBC count, cells x 10^9^ per liter	4.9 (3.9–5.9)	5.9 (3.7–6.2)	7.1 (5.4–8.7)	6.9 (6.5–10)	0.01
Neutrophils	52% (41–58%)	49% (31–55%)	56% (43–64%)	55% (50–65%)	0.5
Lymphocytes	33% (25–46%)	37% (34–53%)	31% (27–41%)	33% (27–41%)	0.6
Monocytes	12% (9–14%)	8% (7–10%)	8% (6–11%)	7% (6–7%)	0.02

ART, antiretroviral therapy; CT, computed tomography; FVC, forced vital capacity. Continuous variables are presented as median values with interquartile ranges (IQR).

a *P*‐value is for comparison across all four groups of subjects, except for HIV‐related variables, which only compare HIV^+^ subjects with low versus preserved DLCO.

b Limit of detection for HIV RNA is 50 copies/mL.

### HIV infection activates diverse pathways in circulating leukocytes

**Figure 1:**
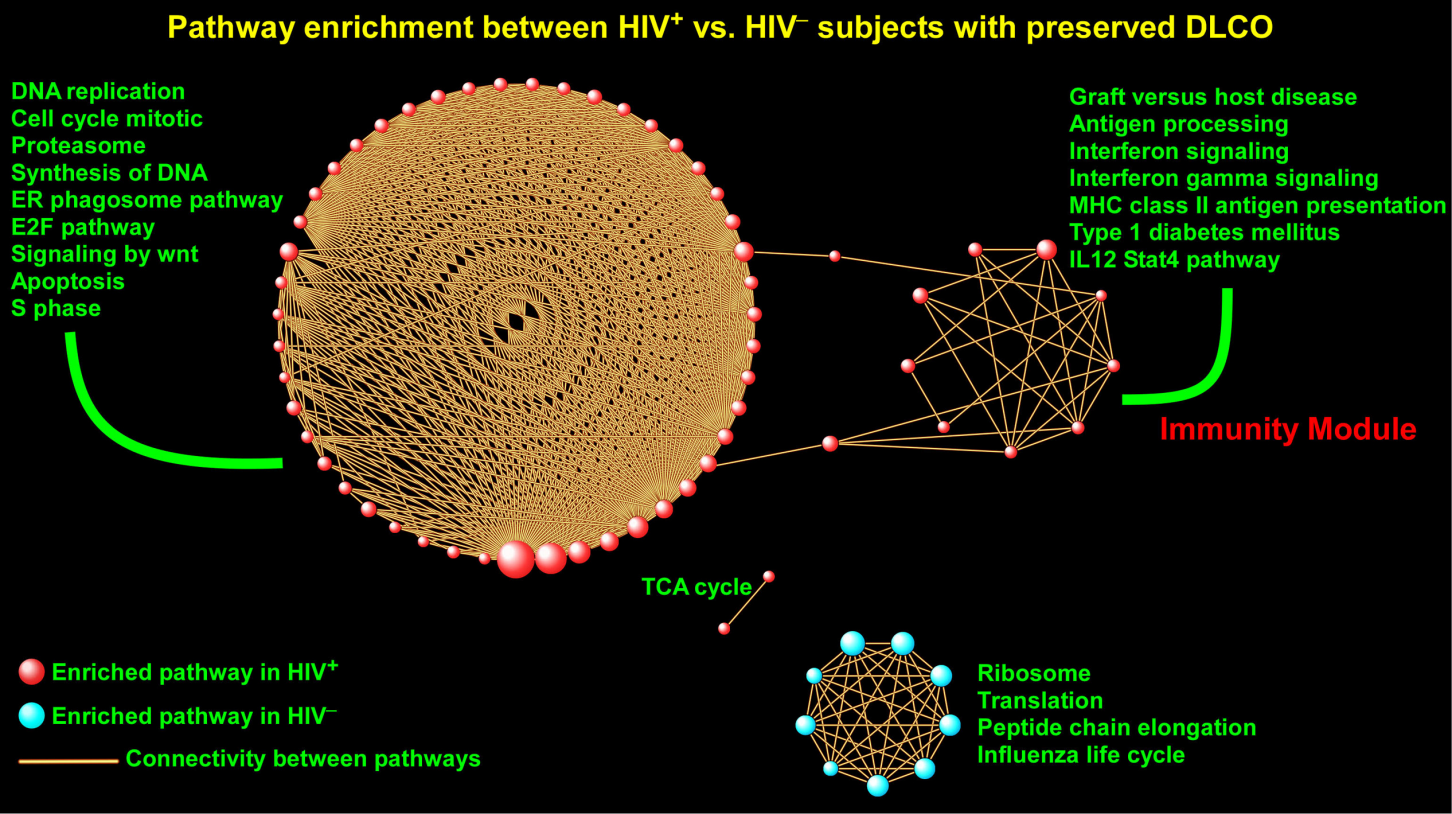
Overview of gene set enrichment analysis between HIV ^+^ and HIV ^–^ subjects with preserved lung diffusing capacity. A gene set was considered enriched for a given phenotype if most of its member genes were up‐regulated in that condition. In the figure, each sphere designates an enriched pathway with red indicating that its member genes were up‐regulated in HIV ^+^ patients and blue indicating that its member genes were up‐regulated in HIV ^–^ subjects. The size of each sphere (i.e., gene set) is proportional to the number of its gene members. Since pathways share many common genes, connectivity lines have been used to link these interpathway relationships and define the topology of the enrichment network. Note that pathways aggregated with each other based on extent of overlap among member genes to form larger modules. Selected gene sets have been labeled and an “Immunity Module” identified. Full list of enriched pathways is available in Table S1.

Since genes do not exert their effects in isolation, but rather cooperate within functionally coherent modules to influence disease susceptibility and progression (Hartwell et al. 1999; Schadt 2009), we opted to analyze the PBL transcriptional profiles using a pathway‐centric approach known as GSEA. Initially we applied GSEA to identify enriched pathways between HIV^+^ versus HIV^–^ subjects with preserved DLCO. Using a strict FDR significance threshold of <0.01 to control multiple hypothesis testing, we identified 61 gene sets that were up‐regulated in PBLs of HIV^+^ patients and only nine pathways up‐regulated in the HIV^–^ subjects (complete list is provided in Table S1). This finding implies that in individuals with normal lung function and gas exchange, HIV infection significantly alters the transcriptional program of circulating leukocytes. We grouped the enriched pathways based on their shared gene membership into larger aggregations known as “modules” that were characterized by common functional attributes. As shown in Figure 1, the largest module was comprised of gene sets up‐regulated in HIV^+^ patients that mapped to processes involved in cell cycle, replication, proteasome, and apoptosis. Another distinct module was populated by immunity‐associated pathways such as interferon signaling, antigen presentation, IL12, and STAT4 pathways. In contrast, the few gene sets enriched in PBLs of HIV^–^ subjects formed a smaller module highlighted by ribosomal, translation, and transcription processes.

### Widespread activation of immuno‐inflammatory programs in HIV^+^ patients with impaired gas exchange

**Figure 2:**
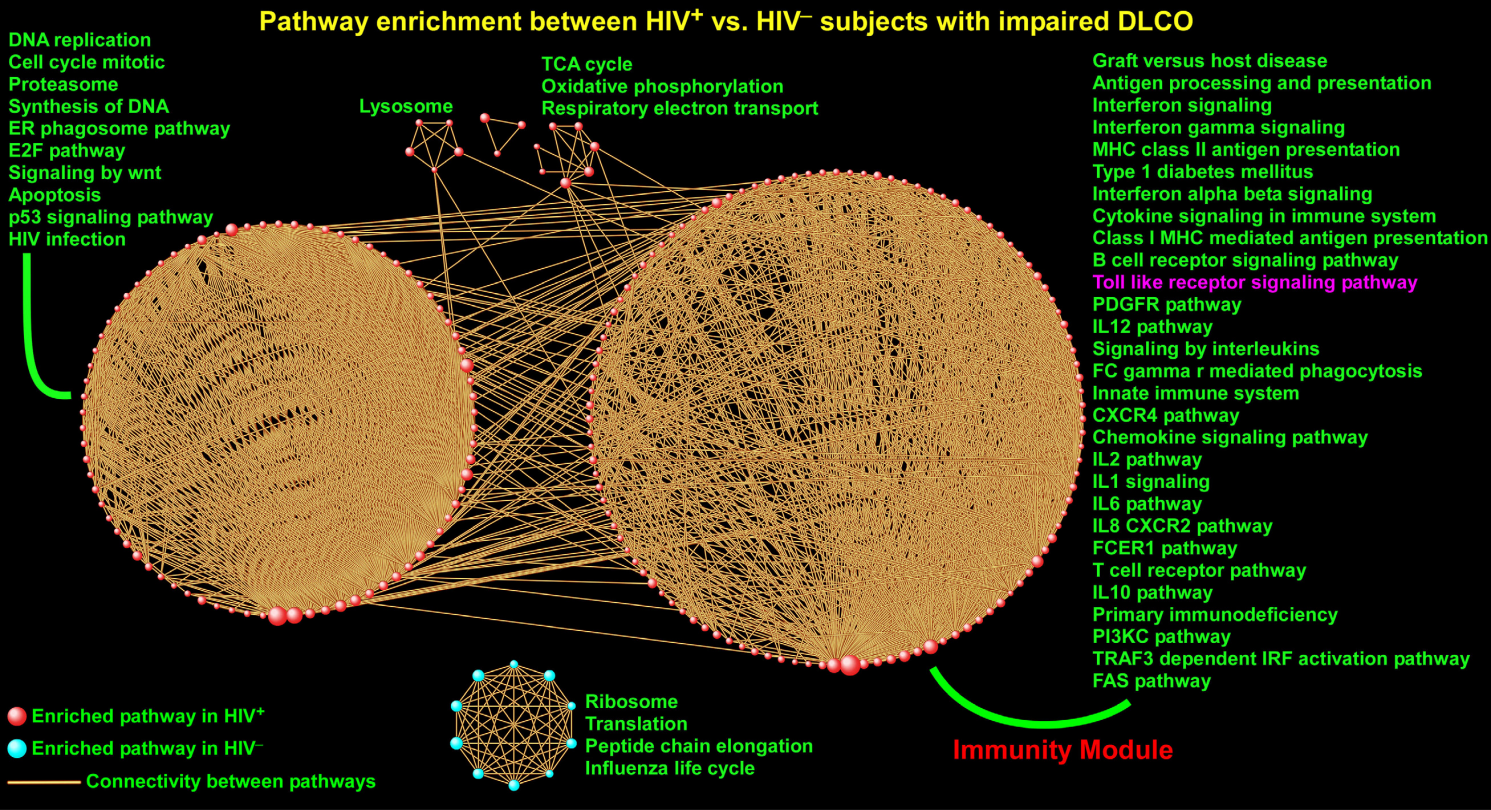
Pathway enrichment analysis between HIV ^+^ and HIV ^–^ subjects with low lung diffusing capacity. Each sphere designates an enriched gene set with red indicating that member genes were up‐regulated in HIV ^+^ patients and blue indicating up‐regulation in HIV ^–^ subjects. The topology of this network has similarities with Figure 1, but notice the much larger module populated by immune and inflammatory pathways enriched in HIV ^+^ patients (“Immunity Module”) including interleukin pathways, innate immunity, and toll‐like receptor signaling. This finding implies that when compared to their DLCO‐matched HIV ^–^ controls, a broader repertoire of immune‐associated pathways and genes are up‐regulated in HIV ^+^ patients with low DLCO compared to HIV ^+^ patients with preserved DLCO (Figure 2 vs. Figure 1). Selected gene sets have been labeled and full list is available in Table S2.

We then investigated the transcriptional consequences of HIV infection in PBLs of patients with low DLCO. We identified 206 up‐regulated pathways in HIV^+^ patients but only 11 up‐regulated gene sets in HIV^–^ subjects (complete list in Table S2). Grouping the enriched pathways into larger modules revealed highly interconnected but distinct gene set assemblies (Fig. 2). Topographically and functionally, there were similarities between these modules and the ones found in HIV^+^ patients with preserved DLCO (Fig. 2 vs. Fig. 1). Several pathways were commonly enriched between the two groups indicating that HIV infection activates a core set of shared transcriptional programs across subjects with and without DLCO impairment. However, we observed that HIV^+^ patients with low DLCO were characterized by aggregation of many gene sets into a much larger immunity module, with activation of multiple cytokine/interleukin pathways, interferon signaling, innate immunity, PDGF receptor pathway, FAS pathway, T‐cell/B‐cell receptor signaling, and toll‐like receptor (TLR) signaling.

**Figure 3:**
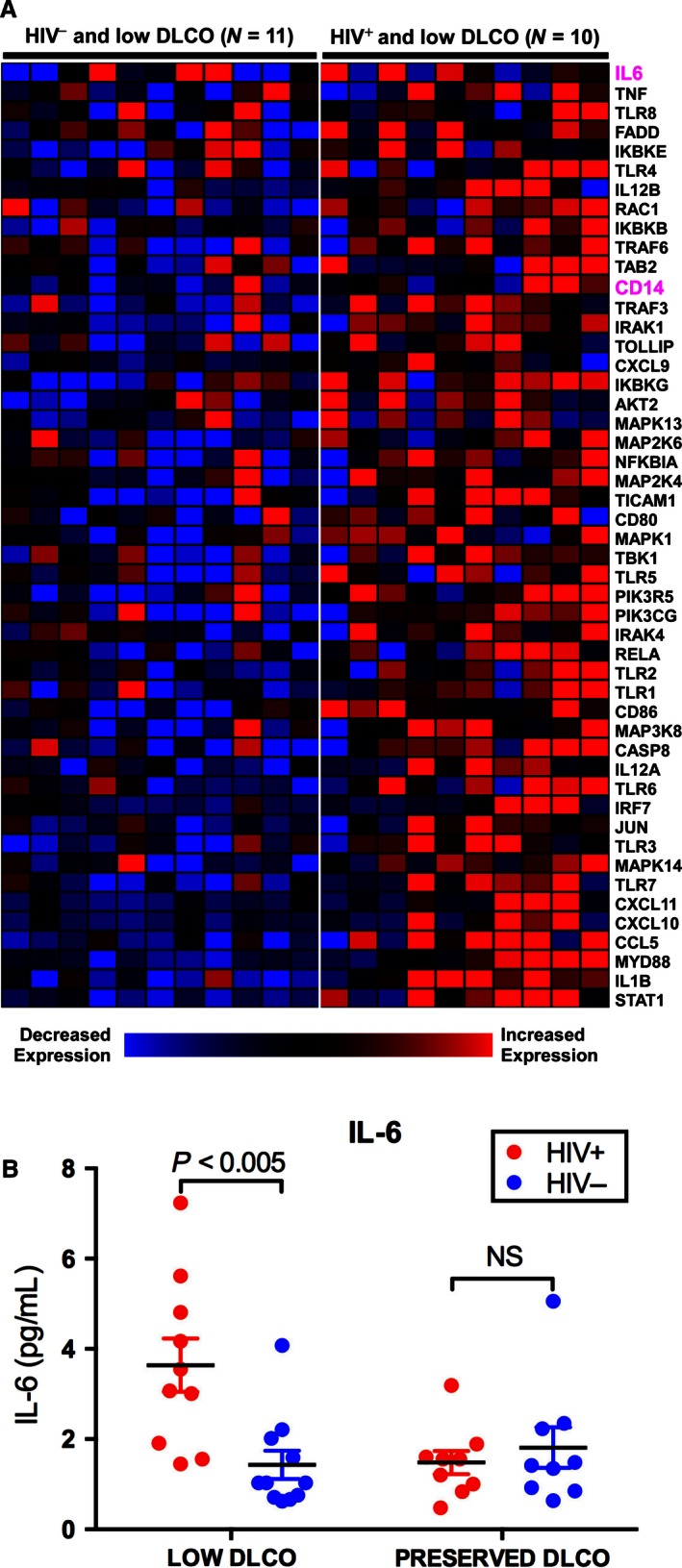
(A) Heat map depiction of the expression profile of toll‐like receptor signaling genes, a representative activated pathway in HIV ^+^ patients with low DLCO. Several members of this pathway such as IL‐6, CD14, and toll‐like receptor (TLR) 4 have been implicated in the pathogenesis of immune activation during chronic HIV infection and may play a role in impaired lung diffusing capacity. (B) Confirmation of up‐regulation of plasma IL‐6 levels in HIV ^+^ patients with low DLCO compared to other subject groups (one‐way analyses of variance (ANOVA) *P*‐value across all groups <0.002, Student's two‐tailed *t*‐test *P*‐values are displayed in Figure).

We investigated the expression profile of “leading edge” genes mapping to TLR signaling cascade – a representative gene set selectively enriched in HIV^+^ patients with impaired gas exchange (Fig. 3A). We measured soluble CD14 (sCD14) and IL‐6, two well‐characterized circulating biomarkers of inflammation (Sandler et al. 2011; Rincon 2012) that were members of this activated pathway. Plasma sCD14 levels were significantly higher in the HIV^+^ patients compared to HIV^–^ subjects (1681 vs. 1367 ng/mL, *P *<* *0.02), but did not differ by DLCO status. This observation is consistent with previous reports associating HIV infection with elevated sCD14 levels as an indicator of increased systemic inflammation and subsequent mortality (Sandler et al. 2011). We found that HIV^+^ subjects with low DLCO had significantly higher plasma IL‐6 levels compared with the other groups, implying an enhanced state of immune activation and inflammation in this subset of patients (Fig. 3B).

These results indicate that while HIV infection alters the transcriptional programming of circulating leukocytes in subjects with preserved gas exchange, this perturbation is much more pronounced in HIV^+^ patients with low DLCO and is highlighted by activation of specific immunological and pro‐inflammatory pathways.

The role of HIV infection in modulating the leukocyte transcriptional response was further explored by performing GSEA in HIV^–^ and HIV^+^ individuals stratified by DLCO. Specifically, in HIV^–^ subjects, we compared those with preserved DLCO versus low DLCO, and in HIV^+^ patients, we performed a similar comparison based on gas exchange status. We found that HIV^–^ individuals with preserved DLCO were characterized by up‐regulation of many immuno‐inflammatory gene sets (254 pathways at FDR <0.01, Table S3), whereas only 17 gene sets were up‐regulated in HIV^–^ subjects with impaired DLCO (Table S3). This observation indicates that in uninfected individuals, reduced diffusing capacity is associated with down‐regulation of pro‐inflammatory transcriptional responses. Intriguingly, when HIV^+^ patients were compared based on their gas exchange status (preserved vs. low DLCO), there were drastically fewer pathways enriched (nine gene sets up‐regulated in HIV^+^ subjects with preserved gas exchange and two pathways up‐regulated in HIV^+^ patients with impaired DLCO, Table S4). Taken together, these findings imply that impaired gas exchange is associated with suppression of immune and inflammatory transcriptional programs in HIV^–^ individuals, but that HIV infection abrogates this response.

Collectively, our data demonstrate that among subjects with impaired gas exchange, HIV infection profoundly influences leukocyte transcriptional response by promoting persistent up‐regulation of diverse pro‐inflammatory and immune‐associated pathways.

## Discussion

With the advent of ART, the life expectancy of HIV^+^ patients has significantly improved and the spectrum of AIDS‐related diseases has changed (Palella et al. 1998; Mocroft et al., 2002). Non‐infectious pulmonary complications are increasingly prevalent in the HIV population, with emphysema and impaired gas exchange among the most common comorbidities in HIV^+^ individuals (Crothers et al. 2006, 2011, 2013; Justice et al. 2006b; Hull et al. 2008). In this study, we compared the transcriptional signatures of circulating leukocytes in HIV^+^ patients versus HIV^−^ subjects as stratified by their pulmonary diffusing capacity status. We found that infection with HIV is associated with systemic activation of immune and inflammatory pathways in circulating leukocytes in subjects with preserved gas exchange. However, HIV^+^ patients with low DLCO had a much more profound and widespread activation of these processes compared to HIV^−^ subjects with similarly reduced DLCO. These results imply that HIV infection in patients with impaired gas transfer is associated with selective enrichment of distinct pro‐inflammatory and immunity programs. Instigation of such immuno‐inflammatory processes in circulating leukocytes may lead to migration of inflammatory cells to the lung and to microvascular dysfunction, thus playing a role in the etiology of low DLCO in HIV^+^ individuals.

The pathogenesis of impaired gas exchange in HIV is unclear, and likely involves diverse processes, including immunologic, apoptotic, proteolytic, and oxidative stress mechanisms (Crothers 2007; Petrache et al. 2008; Kaner et al. 2009). Using a pathway‐focused computational approach, we identified a large repertoire of transcriptional programs in PBLs associated with low DLCO in HIV^+^ patients. Prominent pathways encompassed multiple components of the innate and adaptive immune response including interleukin signaling (IL‐1, IL‐2, IL‐6, IL‐8, IL‐12, IL‐10), interferon signaling, phagocytosis, T‐ and B‐cell receptor signaling, and TLR signaling. To the best of our knowledge, this is the first gene expression analysis of PBLs comparing HIV^+^ versus HIV^−^ subjects as stratified by their pulmonary diffusing capacity status. One study demonstrated up‐regulation of several matrix metalloproteinases (MMPs), including MMP‐2, ‐7, ‐9, and ‐12 in alveolar macrophages from HIV^+^ relative to HIV^–^ patients with early emphysema (Kaner et al. 2009). Similarly, we found enrichment of an MMP gene set in HIV^+^ subjects with low DLCO (Table S2).

Although our experimental design using PBLs did not allow assessment of lung‐specific gene expression, our ability to demonstrate differential gene expression in peripheral blood that correlated with DLCO status suggests that decreased gas exchange is, in part, a reflection of systemic abnormalities. Chronic inflammation and immune activation are characteristic of HIV infection. Impaired barrier function of the gastrointestinal and genital mucosa in HIV infection is thought to facilitate translocation of bacterial products such as lipopolysaccharide (LPS) into the systemic circulation and lead to immune activation via TLR signaling cascade (Brenchley et al. 2006; Nazli et al. 2010; Chege et al. 2011). In addition, coinfections with viruses such as CMV and hepatitis C are likely to contribute to chronic immune activation in HIV^+^ patients. Chronic immune activation has been proposed to be a major contributor to the increased incidence of cardiovascular disease in chronic HIV infection (Malek et al. 2011). Our data suggest that lung dysfunction due to impaired diffusing capacity is another manifestation of chronic immune activation in the setting of HIV infection. This is supported by our prior work that demonstrated elevated levels of soluble CD14, the LPS coreceptor, were associated with emphysema in HIV^+^ patients in a cross‐sectional study (Attia et al. 2014). Furthermore, in our current study we found that plasma IL‐6 levels were significantly increased in HIV^+^ patients with low DLCO compared to HIV^−^ subjects or those with preserved gas exchange, corroborating results from Fitzpatrick et al. who also demonstrated high IL‐6 levels to be associated with low DLCO in those with HIV (Fitzpatrick et al. 2014).

Our study has several limitations, including a modest number of subjects and a relatively heterogeneous population. Although we were limited in our ability to adjust for multiple covariates and confounders within groups, such as differences in CD4 cell count between HIV^+^ subjects with low versus preserved DLCO, our analyses were adequately powered to detect differences in gene expression patterns between groups. To control for a major confounder between groups, we restricted our analysis to current smokers; we also restricted our sample to men. By measuring gene expression levels of all PBLs, we could not assess the relative contribution of leukocyte subsets, and future investigation is required to determine the cell type‐specific transcriptional signals. However, in this pilot project, we applied sophisticated bioinformatics methods to identify, for the first time, pathways whose activation may contribute to gas exchange impairment in HIV^+^ patients. Additional studies are needed to validate our findings and further elucidate the mechanisms by which immune‐activated circulating leukocytes can contribute to pulmonary vascular and gas transfer dysfunction in HIV disease.

In conclusion, we demonstrate that HIV infection in subjects with low DLCO elicits the activation of distinct immunologic and pro‐inflammatory programs in circulating leukocytes, potentially resulting in the migration of inflammatory cells to the lung and to microvascular dysfunction, thus leading to impaired gas exchange in HIV^+^ patients. Our results also identify putative molecular pathways such as the TLR signaling cascade that represent targetable sites for novel treatments aimed at decreasing microbial translocations or suppressing chronic antigenic stimulus from other viral infections that may improve lung function in HIV^+^ individuals.

## Disclaimer

The contents do not represent the views of the U.S. Department of Veterans Affairs or the United States Government.

## Conflict of Interest

None of the authors (KC, IP, CW, PJL, LMS, SAG) have any financial, intellectual, or other conflicts of interest pertaining to this work.

## Supporting information




**Table S1** List of significantly enriched gene sets in PBLs of HIV+ and HIV^−^ subjects with preserved DLCO. FDR <0.01 was used to designate significant enrichment.

**Table S2** List of significantly enriched gene sets in PBLs of HIV+ and HIV^−^ subjects with low DLCO. FDR <0.01 was used to designate significant enrichment.

**Table S3** List of significantly enriched gene sets in PBLs of HIV^−^ negative subjects with preserved versus low DLCO. FDR <0.01 was used to designate significant enrichment.

**Table S4** List of significantly enriched gene sets in PBLs of HIV^+^ subjects with preserved versus low DLCO. FDR <0.01 was used to designate significant enrichment.
