# Supplementary material for: Widespread activation of immunity and pro‐inflammatory programs in peripheral blood leukocytes of HIV‐infected patients with impaired lung gas exchange
Source: Physiol Rep. 2016 Apr 25;4(8):e12756. doi: 10.14814/phy2.12756 (PMC4848721; doi:10.14814/phy2.12756)
Supplement: Supplementary file 1 — Table S1 List of significantly enriched gene sets in PBLs of HIV+ and HIV− subjects with preserved DLCO. FDR <0.01 was used to designate significant enrichment. [file PHY2-4-e12756-s001.pdf]

Supplemental Table 1. List of significantly enriched gene sets in PBLs of HIV+ and HIV- subjects with preserved DLCO.
FDR <0.01 was used to designate significant enrichment.

Gene sets enriched in HIV+ patients with preserved DLCO	Number of genes	FDR
REACTOME_DNA_REPLICATION	188	0
REACTOME_MITOTIC_M_M_G1_PHASES	168	0
PID_AURORA_B_PATHWAY	39	0.00053
REACTOME_ASSEMBLY_OF_THE_PRE_REPLICATIVE_COMPLEX	63	0.00066
REACTOME_CELL_CYCLE_CHECKPOINTS	111	0.00070
REACTOME_CELL_CYCLE	387	0.00072
REACTOME_APC_C_CDC20_MEDIATED_DEGRADATION_OF_MITOTIC_PROTEINS	64	0.00073
KEGG_CITRATE_CYCLE_TCA_CYCLE	30	0.00074
KEGG_ANTIGEN_PROCESSING_AND_PRESENTATION	77	0.00075
REACTOME_REGULATION_OF_MITOTIC_CELL_CYCLE	76	0.00077
REACTOME_INTERFERON_SIGNALING	151	0.00078
REACTOME_ANTIGEN_PROCESSING_CROSS_PRESENTATION	71	0.00079
REACTOME_SYNTHESIS_OF_DNA	90	0.00081
KEGG_GRAFT_VERSUS_HOST_DISEASE	36	0.00088
REACTOME_ORC1_REMOVAL_FROM_CHROMATIN	65	0.00088
REACTOME_ER_PHAGOSOME_PATHWAY	58	0.00096
REACTOME_CDT1_ASSOCIATION_WITH_THE_CDC6_ORC_ORIGIN_COMPLEX	54	0.00098
KEGG_PROTEASOME	44	0.00099
REACTOME_MITOTIC_PROMETAPHASE	86	0.00099
BIOCARTA_ATM_PATHWAY	20	0.00100
REACTOME_SCF_BETA_TRCP_MEDIATED_DEGRADATION_OF_EMI1	49	0.00102
REACTOME_CDK_MEDIATED_PHOSPHORYLATION_AND_REMOVAL_OF_CDC6	46	0.00103
REACTOME_P53_DEPENDENT_G1_DNA_DAMAGE_RESPONSE	53	0.00103
REACTOME_MITOTIC_G1_G1_S_PHASES	130	0.00107
PID_E2F_PATHWAY	74	0.00108
REACTOME_G1_S_TRANSITION	106	0.00110
REACTOME_M_G1_TRANSITION	78	0.00111
KEGG_CELL_CYCLE	123	0.00112
REACTOME_CELL_CYCLE_MITOTIC	303	0.00113
PID_PLK1_PATHWAY	45	0.00122
REACTOME_MHC_CLASS_II_ANTIGEN_PRESENTATION	89	0.00134
REACTOME_VIF_MEDIATED_DEGRADATION_OF_APOBEC3G	49	0.00138
REACTOME_CROSS_PRESENTATION_OF_SOLUBLE_EXOGENOUS_ANTIGENS_ENDOSOMES	46	0.00152
REACTOME_KINESINS	23	0.00153
REACTOME_REGULATION_OF_ORNITHINE_DECARBOXYLASE_ODC	48	0.00157
REACTOME_S_PHASE	106	0.00198
REACTOME_P53_INDEPENDENT_G1_S_DNA_DAMAGE_CHECKPOINT	48	0.00199
REACTOME_APC_C_CDH1_MEDIATED_DEGRADATION_OF_CDC20_AND_OTHER_APC_C_CDH1	63	0.00204
REACTOME_SCFSKP2_MEDIATED_DEGRADATION_OF_P27_P21	53	0.00207
REACTOME_CYCLIN_E_ASSOCIATED_EVENTS_DURING_G1_S_TRANSITION_	62	0.00214
PID_BARD1PATHWAY	29	0.00235
REACTOME_AUTODEGRADATION_OF_THE_E3_UBIQUITIN_LIGASE_COP1	47	0.00237
REACTOME_INTERFERON_GAMMA_SIGNALING	59	0.00243
REACTOME_SIGNALING_BY_WNT	63	0.00245
REACTOME_AUTODEGRADATION_OF_CDH1_BY_CDH1_APC_C	56	0.00245
REACTOME_DESTABILIZATION_OF_MRNA_BY_AUF1_HNRNP_D0	50	0.00329
PID_ATR_PATHWAY	39	0.00361
REACTOME_CITRIC_ACID_CYCLE_TCA_CYCLE	19	0.00375
BIOCARTA_CTL_PATHWAY	13	0.00381
REACTOME_APOPTOSIS	141	0.00441
SA_CASPASE_CASCADE	19	0.00455
PID_IL12_STAT4PATHWAY	33	0.00482
REACTOME_G1_S_SPECIFIC_TRANSCRIPTION	17	0.00583
REACTOME_E2F_MEDIATED_REGULATION_OF_DNA_REPLICATION	33	0.00637
PID_CD8TCRDOWNSTREAMPATHWAY	64	0.00637
KEGG_TYPE_I_DIABETES_MELLITUS	40	0.00639
SA_G1_AND_S_PHASES	15	0.00891

KEGG_ALLOGRAFT_REJECTION	35	0.00920
PID_ERBB1_DOWNSTREAM_PATHWAY	103	0.00929
BIOCARTA_G1_PATHWAY	28	0.00936
PID_RB_1PATHWAY	65	0.00938

**Gene sets enriched in HIV- subjects with preserved DLCO**	**Number of genes**	**FDR**
REACTOME_PEPTIDE_CHAIN_ELONGATION	84	0
KEGG_RIBOSOME	85	0
REACTOME_INFLUENZA_VIRAL_RNA_TRANSCRIPTION_AND_REPLICATION	100	0
REACTOME_3_UTR_MEDIATED_TRANSLATIONAL_REGULATION	103	0
REACTOME_SRP_DEPENDENT_COTRANSLATIONAL_PROTEIN_TARGETING_TO_MEMBRANE	107	0
REACTOME_NONSENSE_MEDIATED_DECAY_ENHANCED_BY_THE_EXON_JUNCTION_COMPLEX	103	0
REACTOME_TRANSLATION	143	0.00016
REACTOME_FORMATION_OF_THE_TERNARY_COMPLEX_AND_SUBSEQUENTLY_THE_43S_COMPLEX	46	0.00034
REACTOME_INFLUENZA_LIFE_CYCLE	134	0.00306
