# Supplementary material for: Widespread activation of immunity and pro‐inflammatory programs in peripheral blood leukocytes of HIV‐infected patients with impaired lung gas exchange
Source: Physiol Rep. 2016 Apr 25;4(8):e12756. doi: 10.14814/phy2.12756 (PMC4848721; doi:10.14814/phy2.12756)
Supplement: Supplementary file 2 — Table S2 List of significantly enriched gene sets in PBLs of HIV+ and HIV− subjects with low DLCO. FDR <0.01 was used to designate significant enrichment. [file PHY2-4-e12756-s002.pdf]

Supplemental Table 2. List of significantly enriched gene sets in PBLs of HIV+ and HIV- subjects with low DLCO.
FDR <0.01 was used to designate significant enrichment.

**Gene sets enriched in HIV+ patients with reduced DLCO**	**Number of genes**	**FDR**
REACTOME_INTERFERON_ALPHA_BETA_SIGNALING	61	0
REACTOME_INTERFERON_SIGNALING	151	0
REACTOME_INTERFERON_GAMMA_SIGNALING	59	0
REACTOME_G1_S_TRANSITION	106	0
REACTOME_MITOTIC_G1_G1_S_PHASES	130	0
REACTOME_CYTOKINE_SIGNALING_IN_IMMUNE_SYSTEM	260	0
REACTOME_ER_PHAGOSOME_PATHWAY	58	0
REACTOME_DNA_REPLICATION	188	0
REACTOME_CELL_CYCLE_CHECKPOINTS	111	0
REACTOME_ANTIGEN_PROCESSING_CROSS_PRESENTATION	71	0
REACTOME_S_PHASE	106	0
REACTOME_MITOTIC_M_M_G1_PHASES	168	0
REACTOME_SYNTHESIS_OF_DNA	90	0
KEGG_PROTEASOME	44	0
REACTOME_E2F_MEDIATED_REGULATION_OF_DNA_REPLICATION	33	0
REACTOME_CROSS_PRESENTATION_OF_SOLUBLE_EXOGENOUS_ANTIGENS_ENDOSOMES	46	0
REACTOME_APC_C_CDC20_MEDIATED_DEGRADATION_OF_MITOTIC_PROTEINS	64	0
REACTOME_CELL_CYCLE_MITOTIC	303	0
REACTOME_CYCLIN_E_ASSOCIATED_EVENTS_DURING_G1_S_TRANSITION_	62	0
REACTOME_M_G1_TRANSITION	78	0
REACTOME_CLASS_I_MHC_MEDIATED_ANTIGEN_PROCESSING_PRESENTATION	231	0
REACTOME_REGULATION_OF_ORNITHINE_DECARBOXYLASE_ODC	48	0
KEGG_LEISHMANIA_INFECTION	68	0
REACTOME_ASSEMBLY_OF_THE_PRE_REPLICATIVE_COMPLEX	63	0
REACTOME_ACTIVATION_OF_NF_KAPPAB_IN_B_CELLS	61	0
REACTOME_CELL_CYCLE	387	0
REACTOME_SIGNALING_BY_THE_B_CELL_RECEPTOR_BCR	121	0
REACTOME_P53_INDEPENDENT_G1_S_DNA_DAMAGE_CHECKPOINT	48	0
REACTOME_CDT1_ASSOCIATION_WITH_THE_CDC6_ORC_ORIGIN_COMPLEX	54	0
REACTOME_SCFSKP2_MEDIATED_DEGRADATION_OF_P27_P21	53	0
REACTOME_REGULATION_OF_MITOTIC_CELL_CYCLE	76	0
REACTOME_SCF_BETA_TRCP_MEDIATED_DEGRADATION_OF_EMI1	49	0
REACTOME_SIGNALING_BY_WNT	63	0
REACTOME_P53_DEPENDENT_G1_DNA_DAMAGE_RESPONSE	53	0
REACTOME_VIF_MEDIATED_DEGRADATION_OF_APOBEC3G	49	0
REACTOME_ORC1_REMOVAL_FROM_CHROMATIN	65	0
REACTOME_CDK_MEDIATED_PHOSPHORYLATION_AND_REMOVAL_OF_CDC6	46	0
REACTOME_TOLL_RECEPTOR_CASCADES	112	0
REACTOME_AUTODEGRADATION_OF_CDH1_BY_CDH1_APC_C	56	0
PID_PDGFRBPATHWAY	126	0
REACTOME_AUTODEGRADATION_OF_THE_E3_UBIQUITIN_LIGASE_COP1	47	0
REACTOME_REGULATION_OF_APOPTOSIS	56	0
REACTOME_APC_C_CDH1_MEDIATED_DEGRADATION_OF_CDC20_AND_OTHER_APC_C_CDH1	63	0
KEGG_ANTIGEN_PROCESSING_AND_PRESENTATION	77	0
REACTOME_DESTABILIZATION_OF_MRNA_BY_AUF1_HNRNP_D0	50	0
BIOCARTA_PROTEASOME_PATHWAY	28	0
REACTOME_REGULATION_OF_MRNA_STABILITY_BY_PROTEINS_THAT_BIND_AU_RICH_ELEMENTS	80	0
PID_IL12_2PATHWAY	62	0
REACTOME_ACTIVATED_TLR4_SIGNALLING	89	0
PID_PLK1_PATHWAY	45	0
PID_AURORA_B_PATHWAY	39	0
REACTOME_G1_S_SPECIFIC_TRANSCRIPTION	17	0
REACTOME_ANTIGEN_PROCESSING_UBIQUITINATION_PROTEASOME_DEGRADATION	195	0
PID_BCR_5PATHWAY	65	0
KEGG_B_CELL_RECEPTOR_SIGNALING_PATHWAY	75	0
REACTOME_APOPTOSIS	141	0

REACTOME_DOWNSTREAM_SIGNALING_EVENTS_OF_B_CELL_RECEPTOR_BCR	92	0.00002
BIOCARTA_MPR_PATHWAY	33	0.00002
PID_ATR_PATHWAY	39	0.00004
REACTOME_SIGNALING_BY_ILS	105	0.00004
REACTOME_SIGNALLING_TO_ERKS	35	0.00005
REACTOME_ANTIGEN_PRESENTATION_FOLDING_ASSEMBLY_AND_PEPTIDE_LOADING_OF_CLASS_I_MHC	20	0.00005
REACTOME_SIGNALLING_TO_RAS	26	0.00006
REACTOME_MYD88_MAL_CASCADE_INITIATED_ON_PLASMA_MEMBRANE	80	0.00007
REACTOME_TRANS_GOLGI_NETWORK_VESICLE_BUDDING	58	0.00010
KEGG_LYSOSOME	120	0.00012
REACTOME_ANTIGEN_ACTIVATES_B_CELL_RECEPTOR_LEADING_TO_GENERATION_OF_SECOND_MESSENGERS	29	0.00023
PID_TXA2PATHWAY	57	0.00023
KEGG_FC_GAMMA_R_MEDIATED_PHAGOCYTOSIS	91	0.00024
BIOCARTA_IL22BP_PATHWAY	16	0.00024
PID_E2F_PATHWAY	74	0.00029
REACTOME_MITOTIC_PROMETAPHASE	86	0.00030
PID_CXCR4_PATHWAY	102	0.00031
REACTOME_INNATE_IMMUNE_SYSTEM	249	0.00032
PID_PRLSIGNALINGEVENTSPATHWAY	23	0.00032
KEGG_APOPTOSIS	87	0.00034
KEGG_TOLL_LIKE_RECEPTOR_SIGNALING_PATHWAY	98	0.00037
KEGG_P53_SIGNALING_PATHWAY	67	0.00052
REACTOME_MHC_CLASS_II_ANTIGEN_PRESENTATION	89	0.00056
KEGG_NOD_LIKE_RECEPTOR_SIGNALING_PATHWAY	62	0.00057
REACTOME_DNA_STRAND_ELONGATION	30	0.00060
PID_GMCSF_PATHWAY	37	0.00061
KEGG_CHEMOKINE_SIGNALING_PATHWAY	179	0.00061
KEGG_GRAFT_VERSUS_HOST_DISEASE	36	0.00061
REACTOME_GOLGI_ASSOCIATED_VESICLE_BIOGENESIS	51	0.00062
KEGG_CELL_CYCLE	123	0.00065
REACTOME_G2_M_CHECKPOINTS	41	0.00065
REACTOME_G0_AND_EARLY_G1	23	0.00066
KEGG_EPITHELIAL_CELL_SIGNALING_IN_HELICOBACTER_PYLORI_INFECTION	68	0.00066
PID_IL2_1PATHWAY	55	0.00066
REACTOME_IMMUNOREGULATORY_INTERACTIONS_BETWEEN_A_LYMPHOID_AND_A_NON_LYMPHOID_CELL	60	0.00069
REACTOME_IL1_SIGNALING	38	0.00070
REACTOME_TRIF_MEDIATED_TLR3_SIGNALING	72	0.00072
REACTOME_SIGNAL_AMPLIFICATION	30	0.00076
KEGG_CYTOSOLIC_DNA_SENSING_PATHWAY	51	0.00077
REACTOME_REGULATION_OF_IFNG_SIGNALING	13	0.00078
REACTOME_MAP_KINASE_ACTIVATION_IN_TLR_CASCADE	49	0.00080
REACTOME_UNFOLDED_PROTEIN_RESPONSE	75	0.00084
KEGG_VIBRIO_CHOLERAE_INFECTION	54	0.00085
PID_ERBB1_DOWNSTREAM_PATHWAY	103	0.00085
KEGG_ONE_CARBON_POOL_BY_FOLATE	17	0.00086
REACTOME_TRAF6_MEDIATED_INDUCTION_OF_NFKB_AND_MAP_KINASES_UPON_TLR7_8_OR_9_ACTIVATION	74	0.00100
REACTOME_HEMOSTASIS	442	0.00100
PID_FOXM1PATHWAY	40	0.00102
PID_CD8TCRPATHWAY	52	0.00102
REACTOME_PLATELET_ACTIVATION_SIGNALING_AND_AGGREGATION	196	0.00109
KEGG_DNA_REPLICATION	36	0.00109
PID_IL6_7PATHWAY	47	0.00113
REACTOME_NFKB_ACTIVATION_THROUGH_FADD_RIP1_PATHWAY_MEDIATED_BY_CASPASE_8_AND10	12	0.00120
BIOCARTA_G2_PATHWAY	24	0.00120
PID_NFKAPPABATYPICALPATHWAY	17	0.00126
REACTOME_ERKS_ARE_INACTIVATED	12	0.00127
REACTOME_CLEAVAGE_OF_GROWING_TRANSCRIPT_IN_THE_TERMINATION_REGION_	43	0.00156
REACTOME_INTRINSIC_PATHWAY_FOR_APOPTOSIS	29	0.00170
PID_RAC1_PATHWAY	51	0.00172
REACTOME_CYCLIN_A_B1_ASSOCIATED_EVENTS_DURING_G2_M_TRANSITION	15	0.00174

PID_FCER1PATHWAY	60	0.00175
REACTOME_PLATELET_SENSITIZATION_BY_LDL	16	0.00179
BIOCARTA_TOLL_PATHWAY	37	0.00181
BIOCARTA_BIOPEPTIDES_PATHWAY	42	0.00195
BIOCARTA_TID_PATHWAY	19	0.00195
BIOCARTA_RB_PATHWAY	13	0.00195
PID_P75NTRPATHWAY	69	0.00196
PID_IL8CXCR2_PATHWAY	34	0.00197
KEGG_NATURAL_KILLER_CELL_MEDIATED_CYTOTOXICITY	127	0.00207
SA_MMP_CYTOKINE_CONNECTION	15	0.00228
REACTOME_P38MAPK_EVENTS	13	0.00238
PID_IFNGPATHWAY	40	0.00239
PID_TCR_PATHWAY	65	0.00242
PID_TOLL_ENDOGENOUS_PATHWAY	25	0.00242
BIOCARTA_IL10_PATHWAY	17	0.00260
REACTOME_GROWTH_HORMONE_RECEPTOR_SIGNALING	24	0.00261
REACTOME_PROCESSING_OF_CAPPED_INTRONLESS_PRE_MRNA	23	0.00263
REACTOME_NFKB_AND_MAP_KINASES_ACTIVATION_MEDIATED_BY_TLR4_SIGNALING_REPERTOIRE	69	0.00263
PID_CDC42_PATHWAY	69	0.00277
PID_MET_PATHWAY	79	0.00285
REACTOME_ACTIVATION_OF_ATR_IN_RESPONSE_TO_REPLICATION_STRESS	35	0.00295
REACTOME_DARPP_32_EVENTS	24	0.00305
REACTOME_ACTIVATION_OF_THE_PRE_REPLICATIVE_COMPLEX	30	0.00318
REACTOME_HIV_INFECTION	191	0.00330
REACTOME_DIABETES_PATHWAYS	124	0.00336
REACTOME_REGULATION_OF_IFNA_SIGNALING	23	0.00341
REACTOME_EXTENSION_OF_TELOMERES	27	0.00343
PID_IL27PATHWAY	26	0.00348
REACTOME_TCA_CYCLE_AND_RESPIRATORY_ELECTRON_TRANSPORT	117	0.00348
PID_INTEGRIN_A4B1_PATHWAY	33	0.00398
PID_P73PATHWAY	79	0.00415
BIOCARTA_SALMONELLA_PATHWAY	12	0.00416
REACTOME_G2_M_DNA_DAMAGE_CHECKPOINT	9	0.00418
REACTOME_RNA_POL_II_TRANSCRIPTION	100	0.00426
KEGG_PRIMARY_IMMUNODEFICIENCY	35	0.00429
REACTOME_ERK_MAPK_TARGETS	21	0.00429
REACTOME_INHIBITION_OF_REPLICATION_INITIATION_OF_DAMAGED_DNA_BY_RB1_E2F1	12	0.00435
KEGG_SNARE_INTERACTIONS_IN_VESICULAR_TRANSPORT	37	0.00438
REACTOME_HOST_INTERACTIONS_OF_HIV_FACTORS	120	0.00452
PID_S1P_S1P4_PATHWAY	14	0.00465
KEGG_TYPE_I_DIABETES_MELLITUS	40	0.00489
REACTOME_MRNA_3_END_PROCESSING	34	0.00489
REACTOME_KINESINS	23	0.00492
BIOCARTA_CHEMICAL_PATHWAY	22	0.00496
PID_AURORA_A_PATHWAY	31	0.00502
REACTOME_CITRIC_ACID_CYCLE_TCA_CYCLE	19	0.00505
REACTOME_SEMA4D_IN_SEMAPHORIN_SIGNALING	28	0.00507
REACTOME_IRAK1_RECRUITS_IKK_COMPLEX	9	0.00508
BIOCARTA_CELLCYCLE_PATHWAY	23	0.00530
KEGG_OXIDATIVE_PHOSPHORYLATION	117	0.00530
BIOCARTA_PML_PATHWAY	17	0.00532
BIOCARTA_MONOCYTE_PATHWAY	11	0.00542
PID_P53DOWNSTREAMPATHWAY	135	0.00572
REACTOME_ARMS_MEDIATED_ACTIVATION	17	0.00575
BIOCARTA_CASPASE_PATHWAY	23	0.00575
REACTOME_RESPIRATORY_ELECTRON_TRANSPORT	65	0.00595
KEGG_NEUROTROPHIN_SIGNALING_PATHWAY	125	0.00596
BIOCARTA_ATM_PATHWAY	20	0.00640
REACTOME_LYSOSOME_VESICLE_BIOGENESIS	22	0.00642
REACTOME_NUCLEOTIDE_BINDING_DOMAIN_LEUCINE_RICH_REPEAT_CONTAINING_RECEPTOR_NLR	44	0.00673

PID_P38ALPHABETAPATHWAY	31	0.00688
REACTOME_MEMBRANE_TRAFFICKING	125	0.00692
PID_EPOPATHWAY	34	0.00703
BIOCARTA_RNA_PATHWAY	10	0.00703
PID_MAPKTRKPATHWAY	34	0.00706
BIOCARTA_MITOCHONDRIA_PATHWAY	21	0.00707
PID_PI3KCIPATHWAY	48	0.00707
REACTOME_MAPK_TARGETS_NUCLEAR_EVENTS_MEDIATED_BY_MAP_KINASES	30	0.00709
REACTOME_NUCLEAR_EVENTS_KINASE_AND_TRANSCRIPTION_FACTOR_ACTIVATION	24	0.00711
BIOCARTA_IL3_PATHWAY	15	0.00716
REACTOME_ACTIVATION_OF_CHAPERONE_GENES_BY_XBP1S	43	0.00716
REACTOME_TRAF3_DEPENDENT_IRF_ACTIVATION_PATHWAY	14	0.00743
REACTOME_SIGNAL_REGULATORY_PROTEIN_SIRP_FAMILY_INTERACTIONS	12	0.00744
REACTOME_THROMBIN_SIGNALLING_THROUGH_PROTEINASE_ACTIVATED_RECEPTORS_PARS	31	0.00753
REACTOME_UNWINDING_OF_DNA	11	0.00757
KEGG_VIRAL_MYOCARDITIS	68	0.00781
REACTOME_ASPARAGINE_N_LINKED_GLYCOSYLATION	78	0.00795
KEGG_PATHOGENIC_ESCHERICHIA_COLI_INFECTION	51	0.00802
REACTOME_GLUCOSE_METABOLISM	64	0.00804
PID_CXCR3PATHWAY	43	0.00822
REACTOME_REGULATED_PROTEOLYSIS_OF_P75NTR	10	0.00824
BIOCARTA_IL2RB_PATHWAY	38	0.00845
REACTOME_RESPIRATORY_ELECTRON_TRANSPORT_ATP_SYNTHESIS_BY_CHEMIOSMOTIC_COUPLING	81	0.00847
REACTOME_FACTORS_INVOLVED_IN_MEGAKARYOCYTE_DEVELOPMENT_AND_PLATELET_PRODUCTION	120	0.00850
PID_P38ALPHABETADOWNSTREAMPATHWAY	38	0.00853
REACTOME_SHC_MEDIATED_SIGNALLING	14	0.00856
ST_TUMOR_NECROSIS_FACTOR_PATHWAY	28	0.00864
KEGG_CITRATE_CYCLE_TCA_CYCLE	30	0.00883
BIOCARTA_FAS_PATHWAY	30	0.00916
PID_PTP1BPATHWAY	50	0.00946
KEGG_ALZHEIMERS_DISEASE	158	0.00964
REACTOME_SEMA4D_INDUCED_CELL_MIGRATION_AND_GROWTH_CONE_COLLAPSE	24	0.00975
ST_FAS_SIGNALING_PATHWAY	63	0.00975
BIOCARTA_HIVNEF_PATHWAY	58	0.00979
BIOCARTA_BLYMPHOCYTE_PATHWAY	11	0.00991
BIOCARTA_CHREBP2_PATHWAY	42	0.00993

**Gene sets enriched in HIV- subjects with reduced DLCO**	**Number of genes**	**FDR**
REACTOME_PEPTIDE_CHAIN_ELONGATION	84	0
KEGG_RIBOSOME	85	0
REACTOME_3_UTR_MEDIATED_TRANSLATIONAL_REGULATION	103	0
REACTOME_INFLUENZA_VIRAL_RNA_TRANSCRIPTION_AND_REPLICATION	100	0
REACTOME_NONSENSE_MEDIATED_DECAY_ENHANCED_BY_THE_EXON_JUNCTION_COMPLEX	103	0
REACTOME_SRP_DEPENDENT_COTRANSLATIONAL_PROTEIN_TARGETING_TO_MEMBRANE	107	0
REACTOME_INFLUENZA_LIFE_CYCLE	134	0
REACTOME_TRANSLATION	143	0
REACTOME_FORMATION_OF_THE_TERNARY_COMPLEX_AND_SUBSEQUENTLY_THE_43S_COMPLEX	46	0
REACTOME_ACTIVATION_OF_THE_MRNA_UPON_BINDING_OF_THE_CAP_BINDING_COMPLEX	54	0
REACTOME_TIGHT_JUNCTION_INTERACTIONS	29	0.00212
