# Supplementary material for: Widespread activation of immunity and pro‐inflammatory programs in peripheral blood leukocytes of HIV‐infected patients with impaired lung gas exchange
Source: Physiol Rep. 2016 Apr 25;4(8):e12756. doi: 10.14814/phy2.12756 (PMC4848721; doi:10.14814/phy2.12756)
Supplement: Supplementary file 3 — Table S3 List of significantly enriched gene sets in PBLs of HIV− negative subjects with preserved versus low DLCO. FDR <0.01 was used to designate significant enrichment. [file PHY2-4-e12756-s003.pdf]

Supplemental Table 4. List of significantly enriched gene sets in PBLs of HIV negative subjects with preserved vs. reduced DLCO.
FDR <0.01 was used to designate significant enrichment.

Gene sets enriched in HIV- subjects with preserved DLCO	Number of genes	FDR
PID_BCR_5PATHWAY	65	0
KEGG_SPLICEOSOME	124	0
REACTOME_SIGNALING_BY_ILS	105	0
KEGG_LEISHMANIA_INFECTION	68	0
REACTOME_CYTOKINE_SIGNALING_IN_IMMUNE_SYSTEM	260	0
BIOCARTA_RELA_PATHWAY	16	0
PID_IL6_7PATHWAY	47	0
BIOCARTA_MAPK_PATHWAY	86	0
BIOCARTA_KERATINOCYTE_PATHWAY	46	0
PID_CD8TCRPATHWAY	52	0
REACTOME_PROCESSING_OF_CAPPED_INTRON_CONTAINING_PRE_MRNA	136	0
BIOCARTA_FMLP_PATHWAY	35	0
REACTOME_ACTIVATED_TLR4_SIGNALLING	89	0.00004
PID_IFNGPATHWAY	40	0.00004
KEGG_TOLL_LIKE_RECEPTOR_SIGNALING_PATHWAY	98	0.00004
ST_TUMOR_NECROSIS_FACTOR_PATHWAY	28	0.00004
BIOCARTA_CDMAC_PATHWAY	16	0.00004
BIOCARTA_CD40_PATHWAY	15	0.00004
REACTOME_REGULATION_OF_MRNA_STABILITY_BY_PROTEINS_THAT_BIND_AU_RICH_ELEMENTS	80	0.00005
REACTOME_MRNA_PROCESSING	154	0.00005
PID_CERAMIDE_PATHWAY	48	0.00005
REACTOME_NUCLEOTIDE_BINDING_DOMAIN_LEUCINE_RICH_REPEAT_CONTAINING_RECEPTOR_NLR_SIGNALING_PA	44	0.00005
BIOCARTA_NFKB_PATHWAY	23	0.00005
PID_FCER1PATHWAY	60	0.00005
PID_MAPKTRKPATHWAY	34	0.00006
KEGG_LYSOSOME	120	0.00006
REACTOME_SIGNALING_BY_THE_B_CELL_RECEPTOR_BCR	121	0.00006
REACTOME_ANTIGEN_ACTIVATES_B_CELL_RECEPTOR_LEADING_TO_GENERATION_OF_SECOND_MESSENGERS	29	0.00007
BIOCARTA_HIVNEF_PATHWAY	58	0.00007
BIOCARTA_CERAMIDE_PATHWAY	22	0.00007
PID_KITPATHWAY	52	0.00007
PID_TCR_PATHWAY	65	0.00008
BIOCARTA_BCR_PATHWAY	34	0.00008
KEGG_NOD_LIKE_RECEPTOR_SIGNALING_PATHWAY	62	0.00009
REACTOME_MRNA_SPLICING	107	0.00009
KEGG_NATURAL_KILLER_CELL_MEDIATED_CYTOTOXICITY	127	0.00010
BIOCARTA_FCER1_PATHWAY	38	0.00010
PID_IL2_1PATHWAY	55	0.00010
KEGG_B_CELL_RECEPTOR_SIGNALING_PATHWAY	75	0.00011
KEGG_EPITHELIAL_CELL_SIGNALING_IN_HELICOBACTER_PYLORI_INFECTION	68	0.00011
REACTOME_TOLL_RECEPTOR_CASCADES	112	0.00011
ST_B_CELL_ANTIGEN_RECEPTOR	39	0.00011
REACTOME_MYD88_MAL_CASCADE_INITIATED_ON_PLASMA_MEMBRANE	80	0.00012
REACTOME_INTERFERON_SIGNALING	151	0.00012
BIOCARTA_TPO_PATHWAY	24	0.00012
KEGG_T_CELL_RECEPTOR_SIGNALING_PATHWAY	107	0.00012
REACTOME_IL_2_SIGNALING	41	0.00012
BIOCARTA_HCMV_PATHWAY	17	0.00012
KEGG_SNARE_INTERACTIONS_IN_VESICULAR_TRANSPORT	37	0.00012
PID_IL1PATHWAY	33	0.00013
REACTOME_SIGNALLING_BY_NGF	210	0.00013
BIOCARTA_TNFR2_PATHWAY	18	0.00013
REACTOME_NOD1_2_SIGNALING_PATHWAY	29	0.00013
SIG_BCR_SIGNALING_PATHWAY	46	0.00013
PID_TELOMERASEPATHWAY	68	0.00013
BIOCARTA_TCR_PATHWAY	43	0.00013
PID_GMCSF_PATHWAY	37	0.00014
REACTOME_ANTIVIRAL_MECHANISM_BY_IFN_STIMULATED_GENES	64	0.00014
BIOCARTA_IL1R_PATHWAY	32	0.00014

BIOCARTA_IL3_PATHWAY	15	0.00014
PID_TRAIL_PATHWAY	28	0.00014
REACTOME_LATENT_INFECTION_OF_HOMO_SAPIENS_WITH_MYCOBACTERIUM_TUBERCULOSIS	31	0.00014
PID_VEGFR1_2_PATHWAY	69	0.00029
PID_AURORA_A_PATHWAY	31	0.00042
REACTOME_SIGNALING_BY_SCF_KIT	75	0.00043
REACTOME_TRANSPORT_OF_MATURE_TRANSCRIPT_TO_CYTOPLASM	53	0.00043
KEGG_NEUROTROPHIN_SIGNALING_PATHWAY	125	0.00046
BIOCARTA_STRESS_PATHWAY	25	0.00049
REACTOME_IL1_SIGNALING	38	0.00050
PID_PDGFRBPATHWAY	126	0.00050
BIOCARTA_EPO_PATHWAY	19	0.00052
BIOCARTA_IL6_PATHWAY	22	0.00054
BIOCARTA_TOLL_PATHWAY	37	0.00055
SA_PTEN_PATHWAY	17	0.00055
REACTOME_TRIF_MEDIATED_TLR3_SIGNALING	72	0.00056
REACTOME_HIV_INFECTION	191	0.00056
REACTOME_SOS_MEDIATED_SIGNALLING	14	0.00057
REACTOME_P75_NTR_RECEPTOR_MEDIATED_SIGNALLING	79	0.00062
PID_TNFPATHWAY	46	0.00063
KEGG_ACUTE_MYELOID_LEUKEMIA	57	0.00066
BIOCARTA_NTHI_PATHWAY	24	0.00067
PID_AVB3_OPN_PATHWAY	31	0.00068
REACTOME_REGULATION_OF_IFNG_SIGNALING	13	0.00069
REACTOME_RNA_POL_II_TRANSCRIPTION	100	0.00069
PID_SMAD2_3PATHWAY	16	0.00070
REACTOME_NFKB_AND_MAP_KINASES_ACTIVATION_MEDIATED_BY_TLR4_SIGNALING_REPERTOIRE	69	0.00070
REACTOME_METABOLISM_OF_RNA	250	0.00070
BIOCARTA_PYK2_PATHWAY	28	0.00071
REACTOME_ACTIVATION_OF_NF_KAPPAB_IN_B_CELLS	61	0.00071
REACTOME_NEGATIVE_REGULATORS_OF_RIG_I_MDA5_SIGNALING	30	0.00072
BIOCARTA_GH_PATHWAY	27	0.00072
BIOCARTA_P38MAPK_PATHWAY	39	0.00073
PID_HIVNEFPATHWAY	35	0.00073
PID_ATM_PATHWAY	34	0.00073
BIOCARTA_TID_PATHWAY	19	0.00074
REACTOME_INNATE_IMMUNE_SYSTEM	249	0.00075
SA_B_CELL_RECEPTOR_COMPLEXES	24	0.00075
PID_NFKAPPABCANONICALPATHWAY	23	0.00076
SIG_PIP3_SIGNALING_IN_B_LYMPHOCYTES	36	0.00076
REACTOME_MRNA_3_END_PROCESSING	34	0.00084
REACTOME_CLEAVAGE_OF_GROWING_TRANSCRIPT_IN_THE_TERMINATION_REGION_	43	0.00088
ST_T_CELL_SIGNAL_TRANSDUCTION	43	0.00095
REACTOME_SIGNALING_BY_WNT	63	0.00096
ST_ERK1_ERK2_MAPK_PATHWAY	32	0.00096
KEGG_FC_EPSILON_RI_SIGNALING_PATHWAY	76	0.00104
REACTOME_SIGNALLING_TO_RAS	26	0.00108
PID_PI3KCIPATHWAY	48	0.00109
BIOCARTA_IL2RB_PATHWAY	38	0.00110
REACTOME_THE_NLRP3_INFLAMMASOME	11	0.00110
PID_CXCR4_PATHWAY	102	0.00111
REACTOME_DESTABILIZATION_OF_MRNA_BY_AUF1_HNRNP_D0	50	0.00112
KEGG_CHEMOKINE_SIGNALING_PATHWAY	179	0.00116
REACTOME_TRIGLYCERIDE_BIOSYNTHESIS	38	0.00131
KEGG_APOPTOSIS	87	0.00140
REACTOME_GLYCOLYSIS	27	0.00146
KEGG_NON_SMALL_CELL_LUNG_CANCER	54	0.00148
PID_TOLL_ENDOGENOUS_PATHWAY	25	0.00149
KEGG_RIG_I_LIKE_RECEPTOR_SIGNALING_PATHWAY	69	0.00150
PID_P38ALPHABETAPATHWAY	31	0.00150
BIOCARTA_GLEEVEC_PATHWAY	23	0.00151
BIOCARTA_EIF4_PATHWAY	24	0.00152
REACTOME_LATE_PHASE_OF_HIV_LIFE_CYCLE	99	0.00152

REACTOME_RIG_I_MDA5_MEDIATED_INDUCTION_OF_IFN_ALPHA_BETA_PATHWAYS	71	0.00152
REACTOME_INFLAMMASOMES	16	0.00157
KEGG_ENDOCYTOSIS	180	0.00158
BIOCARTA_RACCYCD_PATHWAY	26	0.00158
REACTOME_IL_6_SIGNALING	10	0.00160
REACTOME_CELL_DEATH_SIGNALLING_VIA_NRAGE_NRIF_AND_NADE	58	0.00160
REACTOME_FATTY_ACID_TRIACYLGLYCEROL_AND_KETONE_BODY_METABOLISM	164	0.00160
REACTOME_ENDOSOMAL_SORTING_COMPLEX_REQUIRED_FOR_TRANSPORT_ESCRT	25	0.00162
REACTOME_TRAF6_MEDIATED_INDUCTION_OF_NFKB_AND_MAP_KINASES_UPON_TLR7_8_OR_9_ACTIVATION	74	0.00162
BIOCARTA_SODD_PATHWAY	10	0.00164
BIOCARTA_PROTEASOME_PATHWAY	28	0.00165
REACTOME_CTNNB1_PHOSPHORYLATION_CASCADE	16	0.00166
BIOCARTA_RAC1_PATHWAY	23	0.00166
KEGG_CHRONIC_MYELOID_LEUKEMIA	72	0.00167
REACTOME_PLATELET_ACTIVATION_SIGNALING_AND_AGGREGATION	196	0.00170
BIOCARTA_IL2_PATHWAY	22	0.00171
BIOCARTA_SPPA_PATHWAY	22	0.00172
BIOCARTA_CXCR4_PATHWAY	24	0.00177
REACTOME_FATTY_ACYL_COA_BIOSYNTHESIS	18	0.00178
REACTOME_SHC1_EVENTS_IN_EGFR_SIGNALING	15	0.00181
REACTOME_GLUCOSE_METABOLISM	64	0.00186
KEGG_FC_GAMMA_R_MEDIATED_PHAGOCYTOSIS	91	0.00187
REACTOME_INTERFERON_GAMMA_SIGNALING	59	0.00188
REACTOME_HIV_LIFE_CYCLE	112	0.00188
REACTOME_METABOLISM_OF_MRNA	206	0.00189
PID_ERBB1_RECEPTOR_PROXIMAL_PATHWAY	35	0.00189
BIOCARTA_NKCELLS_PATHWAY	19	0.00189
PID_MTOR_4PATHWAY	68	0.00189
REACTOME_PLATELET_SENSITIZATION_BY_LDL	16	0.00190
BIOCARTA_EGF_PATHWAY	31	0.00196
REACTOME_SHC_MEDIATED_SIGNALLING	14	0.00205
REACTOME_DOWNSTREAM_SIGNALING_EVENTS_OF_B_CELL_RECEPTOR_BCR	92	0.00207
REACTOME_MAP_KINASE_ACTIVATION_IN_TLR_CASCADE	49	0.00208
KEGG_ADIPOCYTOKINE_SIGNALING_PATHWAY	66	0.00209
REACTOME_NFKB_ACTIVATION_THROUGH_FADD_RIP1_PATHWAY_MEDIATED_BY_CASPASE_8_AND10	12	0.00210
REACTOME_INTERFERON_ALPHA_BETA_SIGNALING	61	0.00210
SIG_CD40PATHWAYMAP	33	0.00214
REACTOME_HOST_INTERACTIONS_OF_HIV_FACTORS	120	0.00218
REACTOME_SHC_RELATED_EVENTS	15	0.00219
KEGG_LEUKOCYTE_TRANSENDOTHELIAL_MIGRATION	113	0.00220
REACTOME_TCR_SIGNALING	49	0.00222
PID_P53REGULATIONPATHWAY	58	0.00223
BIOCARTA_AT1R_PATHWAY	32	0.00245
REACTOME_GPVI_MEDIATED_ACTIVATION_CASCADE	30	0.00255
REACTOME_IL_3_5_AND_GM_CSF_SIGNALING	43	0.00258
BIOCARTA_ERK5_PATHWAY	17	0.00270
BIOCARTA_PDGF_PATHWAY	32	0.00272
REACTOME_METABOLISM_OF_NON_CODING_RNA	47	0.00296
REACTOME_DEADENYLATION_OF_MRNA	19	0.00297
PID_CXCR3PATHWAY	43	0.00323
SIG_INSULIN_RECEPTOR_PATHWAY_IN_CARDIAC_MYOCYTES	51	0.00324
PID_ERBB1_DOWNSTREAM_PATHWAY	103	0.00346
BIOCARTA_CCR3_PATHWAY	23	0.00348
KEGG_PENTOSE_PHOSPHATE_PATHWAY	27	0.00380
BIOCARTA_CREB_PATHWAY	27	0.00404
PID_FASPATHWAY	38	0.00405
REACTOME_TRANSPORT_OF_RIBONUCLEOPROTEINS_INTO_THE_HOST_NUCLEUS	27	0.00405
REACTOME_SCFSKP2_MEDIATED_DEGRADATION_OF_P27_P21	53	0.00405
PID_ILK_PATHWAY	45	0.00406
REACTOME_ANTIGEN_PROCESSING_CROSS_PRESENTATION	71	0.00412
SA_MMP_CYTOKINE_CONNECTION	15	0.00413
REACTOME_INTERACTIONS_OF_VPR_WITH_HOST_CELLULAR_PROTEINS	32	0.00429
REACTOME_CLASS_I_MHC_MEDIATED_ANTIGEN_PROCESSING_PRESENTATION	231	0.00447

BIOCARTA_GPCR_PATHWAY	34	0.00462
REACTOME_RAF_MAP_KINASE_CASCADE	10	0.00463
REACTOME_INSULIN_RECEPTOR_RECYCLING	22	0.00463
REACTOME_AUTODEGRADATION_OF_CDH1_BY_CDH1_APC_C	56	0.00464
REACTOME_SIGNALING_BY_EGFR_IN_CANCER	105	0.00464
REACTOME_PURINE_SALVAGE	13	0.00466
REACTOME_NGF_SIGNALLING_VIA_TRKA_FROM_THE_PLASMA_MEMBRANE	132	0.00466
REACTOME_SYNTHESIS_OF_VERY_LONG_CHAIN_FATTY_ACYL_COAS	14	0.00471
BIOCARTA_RANKL_PATHWAY	14	0.00486
REACTOME_SHC1_EVENTS_IN_ERBB4_SIGNALING	20	0.00488
PID_RANBP2PATHWAY	11	0.00504
BIOCARTA_IGF1R_PATHWAY	23	0.00505
REACTOME_PERK_REGULATED_GENE_EXPRESSION	27	0.00506
BIOCARTA_ERK_PATHWAY	28	0.00508
REACTOME_SIGNALLING_TO_ERKS	35	0.00513
PID_ERBB2ERBB3PATHWAY	44	0.00520
REACTOME_EXTRINSIC_PATHWAY_FOR_APOPTOSIS	13	0.00546
REACTOME_PROTEOLYTIC_CLEAVAGE_OF_SNARE_COMPLEX_PROTEINS	15	0.00550
BIOCARTA_BIOPEPTIDES_PATHWAY	42	0.00557
KEGG_PROTEASOME	44	0.00560
BIOCARTA_NGF_PATHWAY	18	0.00560
REACTOME_HEMOSTASIS	442	0.00566
BIOCARTA_GRANULOCYTES_PATHWAY	14	0.00566
REACTOME_NRAGE_SIGNALS_DEATH_THROUGH_JNK	43	0.00568
REACTOME_MRNA_SPLICING_MINOR_PATHWAY	42	0.00591
REACTOME_ALPHA_LINOLENIC_ACID_ALA_METABOLISM	11	0.00592
PID_ANTHRAXPATHWAY	17	0.00593
KEGG_PANCREATIC_CANCER	70	0.00628
PID_IL8CXCR2_PATHWAY	34	0.00639
BIOCARTA_IGF1MTOR_PATHWAY	20	0.00662
REACTOME_CD28_DEPENDENT_VAV1_PATHWAY	11	0.00698
BIOCARTA_TFF_PATHWAY	21	0.00704
REACTOME_TCA_CYCLE_AND_RESPIRATORY_ELECTRON_TRANSPORT	117	0.00713
REACTOME_P53_DEPENDENT_G1_DNA_DAMAGE_RESPONSE	53	0.00713
REACTOME_SIGNAL_REGULATORY_PROTEIN_SIRP_FAMILY_INTERACTIONS	12	0.00715
PID_TCRRASPATHWAY	14	0.00716
BIOCARTA_TNFR1_PATHWAY	29	0.00716
BIOCARTA_MONOCYTE_PATHWAY	11	0.00727
BIOCARTA_IGF1_PATHWAY	21	0.00731
BIOCARTA_DEATH_PATHWAY	33	0.00731
REACTOME_TRANSCRIPTION	192	0.00743
BIOCARTA_FAS_PATHWAY	30	0.00745
REACTOME_TRAF6_MEDIATED_NFKB_ACTIVATION	21	0.00774
ST_P38_MAPK_PATHWAY	37	0.00775
BIOCARTA_ETC_PATHWAY	11	0.00779
PID_REG_GR_PATHWAY	82	0.00788
REACTOME_CYCLIN_E_ASSOCIATED_EVENTS_DURING_G1_S_TRANSITION_	62	0.00808
REACTOME_IKK_COMPLEX_RECRUITMENT_MEDIATED_BY_RIP1	9	0.00812
REACTOME_RECRUITMENT_OF_MITOTIC_CENTROSOME_PROTEINS_AND_COMPLEXES	59	0.00821
PID_AR_PATHWAY	60	0.00823
KEGG_ERBB_SIGNALING_PATHWAY	86	0.00824
REACTOME_DOWNSTREAM_TCR_SIGNALING	33	0.00843
PID_P38_MKK3_6PATHWAY	26	0.00846
KEGG_OTHER_GLYCAN_DEGRADATION	16	0.00865
REACTOME_NEP_NS2_INTERACTS_WITH_THE_CELLULAR_EXPORT_MACHINERY	27	0.00922
KEGG_FATTY_ACID_METABOLISM	39	0.00925
BIOCARTA_IL7_PATHWAY	17	0.00925
REACTOME_COSTIMULATION_BY_THE_CD28_FAMILY	61	0.00926
PID_ARF6DOWNSTREAMPATHWAY	14	0.00926
KEGG_ENDOMETRIAL_CANCER	52	0.00927
BIOCARTA_HER2_PATHWAY	22	0.00941
PID_CD40_PATHWAY	31	0.00943
REACTOME_PHOSPHOLIPID_METABOLISM	188	0.00944

PID_HDAC_CLASSI_PATHWAY	65	0.00945
PID_PS1PATHWAY	46	0.00945
REACTOME_RIP_MEDIATED_NFKB_ACTIVATION_VIA_DAI	18	0.00959
REACTOME_TRANSFERRIN_ENDOCYTOSIS_AND_RECYCLING	25	0.00961
BIOCARTA_PPARA_PATHWAY	55	0.00979
REACTOME_METABOLISM_OF_NUCLEOTIDES	69	0.00979

**Gene sets enriched in HIV- subjects with reduced DLCO**	**Number of genes**	**FDR**
REACTOME_OLFACTORY_SIGNALING_PATHWAY	289	0
KEGG_OLFACTORY_TRANSDUCTION	357	0
KEGG_NEUROACTIVE_LIGAND_RECEPTOR_INTERACTION	267	0
REACTOME_NA_CL_DEPENDENT_NEUROTRANSMITTER_TRANSPORTERS	17	0.00013
KEGG_ECM_RECEPTOR_INTERACTION	83	0.00010
REACTOME_AMINE_LIGAND_BINDING_RECEPTORS	37	0.00025
REACTOME_AMINE_COMPOUND_SLC_TRANSPORTERS	27	0.00093
KEGG_DRUG_METABOLISM_CYTOCHROME_P450	61	0.00232
REACTOME_GPCR_LIGAND_BINDING	386	0.00207
KEGG_METABOLISM_OF_XENOBIOTICS_BY_CYTOCHROME_P450	60	0.00349
KEGG_BASAL_CELL_CARCINOMA	55	0.00574
REACTOME_AMINO_ACID_AND_OLIGOPEPTIDE_SLC_TRANSPORTERS	49	0.00630
REACTOME_ACETYLCHOLINE_BINDING_AND_DOWNSTREAM_EVENTS	13	0.00673
REACTOME_NCAM1_INTERACTIONS	39	0.00944
REACTOME_TRANSPORT_OF_INORGANIC_CATIONS_ANIONS_AND_AMINO_ACIDS_OLIGOPEPTIDES	92	0.00921
REACTOME_POTASSIUM_CHANNELS	97	0.00917
REACTOME_PRESYNAPTIC_NICOTINIC_ACETYLCHOLINE_RECEPTORS	12	0.00907
