# Supplementary material for: Widespread activation of immunity and pro‐inflammatory programs in peripheral blood leukocytes of HIV‐infected patients with impaired lung gas exchange
Source: Physiol Rep. 2016 Apr 25;4(8):e12756. doi: 10.14814/phy2.12756 (PMC4848721; doi:10.14814/phy2.12756)
Supplement: Supplementary file 4 — Table S4 List of significantly enriched gene sets in PBLs of HIV+ subjects with preserved versus low DLCO. FDR <0.01 was used to designate significant enrichment. [file PHY2-4-e12756-s004.pdf]

Supplemental Table 4. List of significantly enriched gene sets in PBLs of HIV+ subjects with preserved vs. reduced DLCO.
FDR <0.01 was used to designate significant enrichment.

**Gene sets enriched in HIV+ patients with preserved DLCO**	**Number of genes**	**FDR**
KEGG_SPLICEOSOME	124	0
PID_TELOMERASEPATHWAY	68	0
REACTOME_PROCESSING_OF_CAPPED_INTRON_CONTAINING_PRE_MRNA	136	0
REACTOME_DOWNREGULATION_OF_SMAD2_3_SMAD4_TRANSCRIPTIONAL_ACTIVITY	19	0.00026
REACTOME_MRNA_SPLICING	107	0.00043
BIOCARTA_ECM_PATHWAY	24	0.00183
REACTOME_MRNA_PROCESSING	154	0.00261
BIOCARTA_MAL_PATHWAY	19	0.00298
BIOCARTA_INTEGRIN_PATHWAY	38	0.00945

**Gene sets enriched in HIV+ patients with reduced DLCO**	**Number of genes**	**FDR**
REACTOME_OLFACTORY_SIGNALING_PATHWAY	289	0
KEGG_OLFACTORY_TRANSDUCTION	357	0
